# A new polymorph of dimesitylborinic acid

**DOI:** 10.1107/S1600536808015638

**Published:** 2008-06-07

**Authors:** Matthias Kuhlmann, Thomas Baumgartner, Masood Parvez

**Affiliations:** a Department of Chemistry, The University of Calgary, 2500 University Drive NW, Calgary, Alberta, Canada T2N 1N4

## Abstract

A new polymorph of dimesitylborinic acid (or hydroxy­dimesitylborane), C_18_H_23_BO, showcasing different crystal packing and symmetry, complements the previously reported polymorph [Weese, Bartlett, Murray, Olmstead & Power (1987 ▶). *Inorg. Chem.* **26**, 2409–2413; Entwistle, Batsanov & Marder (2007 ▶). *Acta Cryst.* E**63**, o2639–o2641]. The structure of the title compound contains only one mol­ecule in the asymmetric unit, and no O—H⋯O inter­actions are observed. However, mol­ecules are linked by weak inter­molecuar O—H⋯π(arene) inter­actions to form centrosymmetric dimers.

## Related literature

For related literature, see: Cornet *et al.* (2003 ▶); Entwistle *et al.* (2007 ▶); Fraenk *et al.* (2001 ▶); Kuhlmann *et al.* (2008 ▶); Weese *et al.* (1987 ▶).
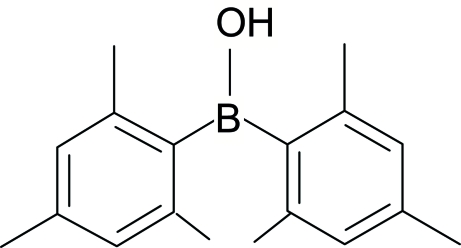


## Experimental

### 

#### Crystal data

C_18_H_23_BO
*M* *_r_* = 266.17
Monoclinic, 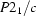

*a* = 8.942 (4) Å
*b* = 8.801 (2) Å
*c* = 19.947 (8) Å
β = 97.800 (16)°
*V* = 1555.3 (10) Å^3^
*Z* = 4
Mo *K*α radiation
μ = 0.07 mm^−1^
*T* = 173 (2) K
0.32 × 0.24 × 0.12 mm

#### Data collection

Nonius KappaCCD diffractometer
Absorption correction: multi-scan (*SORTAV*; Blessing, 1997 ▶) *T*_min_ = 0.979, *T*_max_ = 0.992
6119 measured reflections
3542 independent reflections
2728 reflections with *I* > 2σ(*I*)
*R*_int_ = 0.023

#### Refinement

*R*[*F* ^2^ > 2σ(*F* ^2^)] = 0.047
*wR*(*F* ^2^) = 0.134
*S* = 0.91
3542 reflections
188 parameters
H atoms treated by a mixture of independent and constrained refinement
Δρ_max_ = 0.26 e Å^−3^
Δρ_min_ = −0.19 e Å^−3^

### 

Data collection: *COLLECT* (Hooft, 1998 ▶); cell refinement: *DENZO* (Otwinowski & Minor, 1997 ▶); data reduction: *SCALEPACK* (Otwinowski & Minor, 1997 ▶); program(s) used to solve structure: *SHELXS97* (Sheldrick, 2008 ▶); program(s) used to refine structure: *SHELXL97* (Sheldrick, 2008 ▶); molecular graphics: *ORTEP-3 for Windows* (Farrugia, 1997 ▶); software used to prepare material for publication: *SHELXL97*.

## Supplementary Material

Crystal structure: contains datablocks global, I. DOI: 10.1107/S1600536808015638/om2237sup1.cif

Structure factors: contains datablocks I. DOI: 10.1107/S1600536808015638/om2237Isup2.hkl

Additional supplementary materials:  crystallographic information; 3D view; checkCIF report

## Figures and Tables

**Table 1:** Hydrogen-bond geometry (Å, °) *Cg* is the centroid of the C11–C16 phenyl ring.

*D*—H⋯*A*	*D*—H	H⋯*A*	*D*⋯*A*	*D*—H⋯*A*
O1—H1⋯*Cg*^i^	0.84 (2)	2.83 (2)	3.523 (2)	141 (2)

Symmetry code: (i) 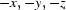.

**Fig. 1.**
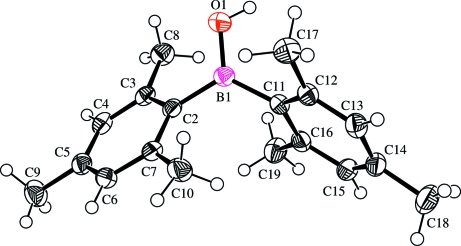
A view of the title compound with displacement ellipsoids plotted at the 50% probability level. Only three H atoms on C9 and C18 are shown.

**Fig. 2.**
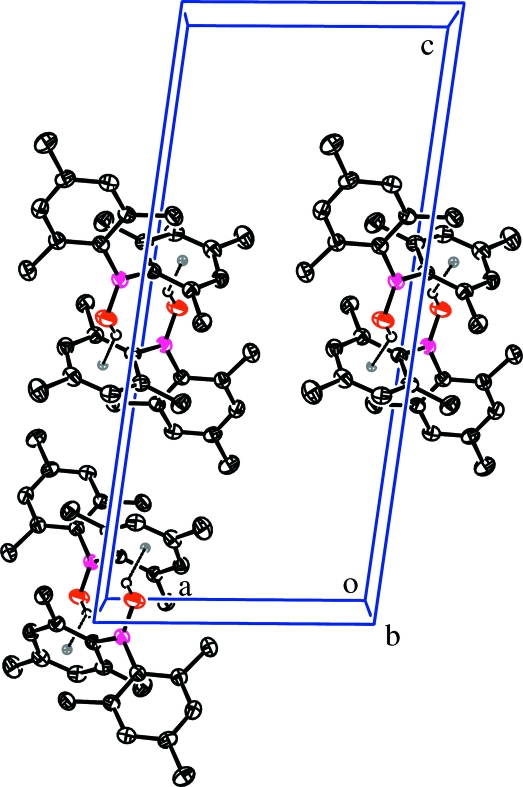
Unit cell packing as viewed down the b direction showing intermolecular O—H···π(arene) interactions with dashed lines.

## supplementary crystallographic information

### Comment

In the course of our studies on the synthesis and photophysical properties of boron-functionalized dithieno[3,2-b;2',3'-d]phospholes (Kuhlmann *et al.,* 2008), we obtained a new polymorph of the title compound. It crystallizes in space group P2_1_/c, which is distinct from the previously reported space group C2/c (Weese *et al.,* 1987; Entwistle *et al.,* 2007).

The structure is composed of unique molecules (Fig. 1) separated by normal van der Waals distances. The mean planes of the phenyl rings are inclined at approximately right angles (86.09 (4)°) with respect to each other. The structure is devoid of any classical hydrogen bonding despite the presence of a hydroxyl group. The previously reported polymorph crystallized as tetramers due to hydrogen bonding involving the OH groups (Weese *et al.,* 1987). There are no indications of π-π stacking interactions between the phenyl groups of the symmetry related molecules in the title compound. However, the hydroxyl group is oriented towards a phenyl ring (C11-C16), thus linking the molecules by rather weak intermolecular O—H···π(arene) interactions to form centrosymmetric dimers about inversion centers (Fig. 2). The boron centers are planar with C2—B1—C11 angle 123.21 (12)°, compared to a wider corresponding angle of 126.0 (4)° reported in the other polymorph (Weese *et al.,* 1987). The structures of trifluoromethyl analogues have also been reported, and they exhibit a similar molecular conformation (Fraenk *et al.,* 2001; Cornet *et al.,* 2003).

### Experimental

The title compound was obtained as hydrolysis by-product in the synthesis of 2-(dimetsitylboryl)-5-phenyl-dithieno[3,2 - b;2',3'-d]phosphole, during the recrystallization of the sample from a concentrated pentane solution at 277 K.

### Refinement

The H-atoms bonded to C9 and C18 were disordered into two sets of methyl groups; the HFIX 123 command in *SHELXTL* (Sheldrick, 2008) was used to model these methyl groups. H-atoms bonded to C-atoms were included at geometrically idealized positions and refined in the riding-model approximation with the following constraints: C—H distances were set to 0.95 Å (aryl) and 0.98 Å (methyl) with *U*_iso_(H) = 1.2*U*_eq_(C). The position of the H-atom bonded to O1 was determined from a difference map and was allowed to refine with *U*_iso_ = 1.2 times *U*_eq_ of O1.

### Crystal data

**Table:**

C_18_H_23_BO	*F*_000_ = 576
*M**_r_* = 266.17	*D*_x_ = 1.137 Mg m^−^^3^
Monoclinic, *P*2_1_/*c*	Mo *K*α radiation λ = 0.71073 Å
Hall symbol: -P 2ybc	Cell parameters from 6119 reflections
*a* = 8.942 (4) Å	θ = 3.3–27.5º
*b* = 8.801 (2) Å	µ = 0.07 mm^−^^1^
*c* = 19.947 (8) Å	*T* = 173 (2) K
β = 97.800 (16)º	Block, yellow
*V* = 1555.3 (10) Å^3^	0.32 × 0.24 × 0.12 mm
*Z* = 4	

### Data collection

**Table:**

Nonius KappaCCD diffractometer	3542 independent reflections
Radiation source: fine-focus sealed tube	2728 reflections with *I* > 2σ(*I*)
Monochromator: graphite	*R*_int_ = 0.023
*T* = 173(2) K	θ_max_ = 27.5º
ω and φ scans	θ_min_ = 3.3º
Absorption correction: multi-scan(SORTAV; Blessing, 1997)	*h* = −11→11
*T*_min_ = 0.979, *T*_max_ = 0.992	*k* = −11→11
6119 measured reflections	*l* = −25→25

### Refinement

**Table:**

Refinement on *F*^2^	Secondary atom site location: difference Fourier map
Least-squares matrix: full	Hydrogen site location: inferred from neighbouring sites
*R*[*F*^2^ > 2σ(*F*^2^)] = 0.047	H atoms treated by a mixture of independent and constrained refinement
*wR*(*F*^2^) = 0.134	*w* = 1/[σ^2^(*F*_o_^2^) + (0.072*P*)^2^ + 0.71*P*] where *P* = (*F*_o_^2^ + 2*F*_c_^2^)/3
*S* = 0.91	(Δ/σ)_max_ < 0.001
3542 reflections	Δρ_max_ = 0.26 e Å^−^^3^
188 parameters	Δρ_min_ = −0.19 e Å^−^^3^
Primary atom site location: structure-invariant direct methods	Extinction correction: none

### Special details

**Table:**

Geometry. All e.s.d.'s (except the e.s.d. in the dihedral angle between two l.s. planes) are estimated using the full covariance matrix. The cell e.s.d.'s are taken into account individually in the estimation of e.s.d.'s in distances, angles and torsion angles; correlations between e.s.d.'s in cell parameters are only used when they are defined by crystal symmetry. An approximate (isotropic) treatment of cell e.s.d.'s is used for estimating e.s.d.'s involving l.s. planes.
Refinement. Refinement of *F*^2^ against ALL reflections. The weighted *R*-factor *wR* and goodness of fit *S* are based on *F*^2^, conventional *R*-factors *R* are based on *F*, with *F* set to zero for negative *F*^2^. The threshold expression of *F*^2^ > σ(*F*^2^) is used only for calculating *R*-factors(gt) *etc*. and is not relevant to the choice of reflections for refinement. *R*-factors based on *F*^2^ are statistically about twice as large as those based on *F*, and *R*- factors based on ALL data will be even larger.

### Fractional atomic coordinates and isotropic or equivalent isotropic displacement parameters (Å^2^)

**Table:**

	*x*	*y*	*z*	*U*_iso_*/*U*_eq_	Occ. (<1)
O1	0.11790 (13)	−0.31566 (13)	−0.00945 (5)	0.0401 (3)	
H1	0.076 (2)	−0.251 (2)	−0.0366 (10)	0.048*	
B1	0.08930 (17)	−0.28800 (17)	0.05445 (8)	0.0269 (3)	
C2	0.17055 (15)	−0.39649 (14)	0.11108 (6)	0.0255 (3)	
C3	0.32785 (15)	−0.42311 (15)	0.11607 (7)	0.0278 (3)	
C4	0.39909 (15)	−0.51699 (15)	0.16679 (7)	0.0309 (3)	
H4	0.5049	−0.5324	0.1698	0.037*	
C5	0.32018 (16)	−0.58890 (15)	0.21314 (7)	0.0316 (3)	
C6	0.16562 (16)	−0.56426 (15)	0.20763 (7)	0.0310 (3)	
H6	0.1095	−0.6138	0.2384	0.037*	
C7	0.09009 (15)	−0.46888 (15)	0.15823 (7)	0.0276 (3)	
C8	0.42383 (17)	−0.35135 (18)	0.06779 (8)	0.0374 (4)	
H8A	0.5308	−0.3633	0.0857	0.045*	
H8B	0.3995	−0.2430	0.0628	0.045*	
H8C	0.4034	−0.4013	0.0236	0.045*	
C9	0.3997 (2)	−0.68880 (19)	0.26809 (8)	0.0446 (4)	
H9A	0.5077	−0.6926	0.2641	0.067*	0.50
H9B	0.3574	−0.7916	0.2636	0.067*	0.50
H9C	0.3858	−0.6472	0.3124	0.067*	0.50
H9D	0.3262	−0.7283	0.2960	0.067*	0.50
H9E	0.4765	−0.6294	0.2965	0.067*	0.50
H9F	0.4481	−0.7737	0.2476	0.067*	0.50
C10	−0.07788 (16)	−0.44837 (18)	0.15670 (8)	0.0364 (3)	
H10A	−0.1137	−0.5144	0.1907	0.044*	
H10B	−0.1295	−0.4752	0.1117	0.044*	
H10C	−0.0995	−0.3422	0.1666	0.044*	
C11	−0.02005 (15)	−0.15448 (14)	0.06966 (7)	0.0265 (3)	
C12	−0.17091 (15)	−0.15062 (15)	0.03816 (7)	0.0286 (3)	
C13	−0.27017 (16)	−0.04211 (16)	0.05784 (7)	0.0320 (3)	
H13	−0.3721	−0.0420	0.0369	0.038*	
C14	−0.22404 (17)	0.06574 (16)	0.10723 (7)	0.0337 (3)	
C15	−0.07360 (17)	0.06623 (15)	0.13554 (7)	0.0330 (3)	
H15	−0.0392	0.1424	0.1677	0.040*	
C16	0.02899 (16)	−0.04183 (15)	0.11824 (7)	0.0292 (3)	
C17	−0.23031 (17)	−0.26522 (18)	−0.01535 (8)	0.0367 (3)	
H17A	−0.3408	−0.2611	−0.0228	0.044*	
H17B	−0.1976	−0.3674	−0.0002	0.044*	
H17C	−0.1910	−0.2415	−0.0577	0.044*	
C18	−0.3345 (2)	0.17870 (19)	0.12935 (10)	0.0471 (4)	
H18A	−0.4347	0.1610	0.1040	0.071*	0.50
H18B	−0.3013	0.2821	0.1206	0.071*	0.50
H18C	−0.3392	0.1664	0.1778	0.071*	0.50
H18D	−0.2821	0.2453	0.1643	0.071*	0.50
H18E	−0.4155	0.1242	0.1477	0.071*	0.50
H18F	−0.3776	0.2399	0.0904	0.071*	0.50
C19	0.18953 (18)	−0.03689 (18)	0.15248 (8)	0.0405 (4)	
H19A	0.2080	0.0600	0.1763	0.049*	
H19B	0.2584	−0.0468	0.1185	0.049*	
H19C	0.2068	−0.1207	0.1850	0.049*	

### Atomic displacement parameters (Å^2^)

**Table:**

	*U*^11^	*U*^22^	*U*^33^	*U*^12^	*U*^13^	*U*^23^
O1	0.0500 (7)	0.0443 (6)	0.0276 (5)	0.0133 (5)	0.0115 (5)	0.0046 (4)
B1	0.0270 (7)	0.0273 (7)	0.0274 (8)	−0.0023 (6)	0.0068 (6)	−0.0004 (6)
C2	0.0284 (7)	0.0225 (6)	0.0259 (6)	0.0005 (5)	0.0056 (5)	−0.0035 (5)
C3	0.0298 (7)	0.0247 (6)	0.0298 (7)	−0.0015 (5)	0.0066 (5)	−0.0061 (5)
C4	0.0276 (7)	0.0282 (7)	0.0359 (8)	0.0022 (5)	0.0012 (6)	−0.0078 (5)
C5	0.0378 (8)	0.0267 (7)	0.0286 (7)	0.0017 (6)	−0.0012 (6)	−0.0035 (5)
C6	0.0381 (8)	0.0282 (7)	0.0270 (7)	−0.0007 (6)	0.0059 (6)	0.0002 (5)
C7	0.0309 (7)	0.0264 (6)	0.0260 (6)	0.0010 (5)	0.0060 (5)	−0.0030 (5)
C8	0.0321 (7)	0.0411 (8)	0.0409 (8)	−0.0018 (6)	0.0119 (6)	−0.0009 (6)
C9	0.0470 (9)	0.0417 (9)	0.0418 (9)	0.0063 (7)	−0.0054 (7)	0.0067 (7)
C10	0.0331 (8)	0.0413 (8)	0.0368 (8)	0.0021 (6)	0.0124 (6)	0.0079 (6)
C11	0.0302 (7)	0.0247 (6)	0.0251 (6)	0.0009 (5)	0.0055 (5)	0.0032 (5)
C12	0.0308 (7)	0.0260 (6)	0.0290 (7)	−0.0008 (5)	0.0042 (5)	0.0032 (5)
C13	0.0289 (7)	0.0315 (7)	0.0358 (8)	0.0019 (5)	0.0048 (6)	0.0051 (6)
C14	0.0380 (8)	0.0271 (7)	0.0380 (8)	0.0059 (6)	0.0122 (6)	0.0043 (6)
C15	0.0431 (8)	0.0249 (7)	0.0313 (7)	0.0009 (6)	0.0057 (6)	−0.0032 (5)
C16	0.0341 (7)	0.0255 (6)	0.0278 (7)	0.0004 (5)	0.0032 (6)	0.0029 (5)
C17	0.0350 (8)	0.0383 (8)	0.0360 (8)	−0.0045 (6)	0.0012 (6)	−0.0047 (6)
C18	0.0457 (9)	0.0391 (9)	0.0590 (11)	0.0129 (7)	0.0168 (8)	−0.0020 (7)
C19	0.0391 (8)	0.0373 (8)	0.0424 (8)	0.0021 (6)	−0.0044 (7)	−0.0085 (7)

### Geometric parameters (Å, °)

**Table:**

O1—B1	1.3556 (19)	C10—H10B	0.9800
O1—H1	0.84 (2)	C10—H10C	0.9800
B1—C2	1.579 (2)	C11—C12	1.409 (2)
B1—C11	1.584 (2)	C11—C16	1.4139 (19)
C2—C7	1.4113 (19)	C12—C13	1.3958 (19)
C2—C3	1.4161 (19)	C12—C17	1.512 (2)
C3—C4	1.3918 (19)	C13—C14	1.390 (2)
C3—C8	1.512 (2)	C13—H13	0.9500
C4—C5	1.389 (2)	C14—C15	1.386 (2)
C4—H4	0.9500	C14—C18	1.509 (2)
C5—C6	1.388 (2)	C15—C16	1.397 (2)
C5—C9	1.506 (2)	C15—H15	0.9500
C6—C7	1.3968 (19)	C16—C19	1.504 (2)
C6—H6	0.9500	C17—H17A	0.9800
C7—C10	1.509 (2)	C17—H17B	0.9800
C8—H8A	0.9800	C17—H17C	0.9800
C8—H8B	0.9800	C18—H18A	0.9800
C8—H8C	0.9800	C18—H18B	0.9800
C9—H9A	0.9800	C18—H18C	0.9800
C9—H9B	0.9800	C18—H18D	0.9800
C9—H9C	0.9800	C18—H18E	0.9800
C9—H9D	0.9800	C18—H18F	0.9800
C9—H9E	0.9800	C19—H19A	0.9800
C9—H9F	0.9800	C19—H19B	0.9800
C10—H10A	0.9800	C19—H19C	0.9800
			
B1—O1—H1	111.0 (13)	H10A—C10—H10C	109.5
O1—B1—C2	115.90 (12)	H10B—C10—H10C	109.5
O1—B1—C11	120.89 (12)	C12—C11—C16	118.54 (12)
C2—B1—C11	123.21 (12)	C12—C11—B1	120.73 (12)
C7—C2—C3	118.02 (12)	C16—C11—B1	120.62 (12)
C7—C2—B1	121.54 (12)	C13—C12—C11	119.86 (13)
C3—C2—B1	120.43 (12)	C13—C12—C17	118.63 (13)
C4—C3—C2	120.10 (12)	C11—C12—C17	121.47 (12)
C4—C3—C8	117.87 (13)	C14—C13—C12	121.86 (13)
C2—C3—C8	122.03 (13)	C14—C13—H13	119.1
C5—C4—C3	122.04 (13)	C12—C13—H13	119.1
C5—C4—H4	119.0	C15—C14—C13	117.97 (13)
C3—C4—H4	119.0	C15—C14—C18	121.08 (14)
C6—C5—C4	117.78 (13)	C13—C14—C18	120.95 (14)
C6—C5—C9	121.02 (13)	C14—C15—C16	122.05 (13)
C4—C5—C9	121.20 (14)	C14—C15—H15	119.0
C5—C6—C7	122.04 (13)	C16—C15—H15	119.0
C5—C6—H6	119.0	C15—C16—C11	119.59 (13)
C7—C6—H6	119.0	C15—C16—C19	119.10 (13)
C6—C7—C2	120.00 (13)	C11—C16—C19	121.30 (12)
C6—C7—C10	118.09 (12)	C12—C17—H17A	109.5
C2—C7—C10	121.91 (12)	C12—C17—H17B	109.5
C3—C8—H8A	109.5	H17A—C17—H17B	109.5
C3—C8—H8B	109.5	C12—C17—H17C	109.5
H8A—C8—H8B	109.5	H17A—C17—H17C	109.5
C3—C8—H8C	109.5	H17B—C17—H17C	109.5
H8A—C8—H8C	109.5	C14—C18—H18A	109.5
H8B—C8—H8C	109.5	C14—C18—H18B	109.5
C5—C9—H9A	109.5	H18A—C18—H18B	109.5
C5—C9—H9B	109.5	C14—C18—H18C	109.5
H9A—C9—H9B	109.5	H18A—C18—H18C	109.5
C5—C9—H9C	109.5	H18B—C18—H18C	109.5
H9A—C9—H9C	109.5	C14—C18—H18D	109.5
H9B—C9—H9C	109.5	H18A—C18—H18D	141.1
C5—C9—H9D	109.5	H18B—C18—H18D	56.3
H9A—C9—H9D	141.1	H18C—C18—H18D	56.3
H9B—C9—H9D	56.3	C14—C18—H18E	109.5
H9C—C9—H9D	56.3	H18A—C18—H18E	56.3
C5—C9—H9E	109.5	H18B—C18—H18E	141.1
H9A—C9—H9E	56.3	H18C—C18—H18E	56.3
H9B—C9—H9E	141.1	H18D—C18—H18E	109.5
H9C—C9—H9E	56.3	C14—C18—H18F	109.5
H9D—C9—H9E	109.5	H18A—C18—H18F	56.3
C5—C9—H9F	109.5	H18B—C18—H18F	56.3
H9A—C9—H9F	56.3	H18C—C18—H18F	141.1
H9B—C9—H9F	56.3	H18D—C18—H18F	109.5
H9C—C9—H9F	141.1	H18E—C18—H18F	109.5
H9D—C9—H9F	109.5	C16—C19—H19A	109.5
H9E—C9—H9F	109.5	C16—C19—H19B	109.5
C7—C10—H10A	109.5	H19A—C19—H19B	109.5
C7—C10—H10B	109.5	C16—C19—H19C	109.5
H10A—C10—H10B	109.5	H19A—C19—H19C	109.5
C7—C10—H10C	109.5	H19B—C19—H19C	109.5
			
O1—B1—C2—C7	−130.25 (14)	O1—B1—C11—C12	58.47 (18)
C11—B1—C2—C7	49.51 (18)	C2—B1—C11—C12	−121.28 (14)
O1—B1—C2—C3	50.12 (18)	O1—B1—C11—C16	−125.45 (15)
C11—B1—C2—C3	−130.12 (14)	C2—B1—C11—C16	54.80 (18)
C7—C2—C3—C4	−0.66 (18)	C16—C11—C12—C13	−3.41 (19)
B1—C2—C3—C4	178.98 (12)	B1—C11—C12—C13	172.75 (12)
C7—C2—C3—C8	179.76 (12)	C16—C11—C12—C17	178.63 (13)
B1—C2—C3—C8	−0.60 (19)	B1—C11—C12—C17	−5.21 (19)
C2—C3—C4—C5	0.9 (2)	C11—C12—C13—C14	1.3 (2)
C8—C3—C4—C5	−179.46 (13)	C17—C12—C13—C14	179.29 (13)
C3—C4—C5—C6	−0.1 (2)	C12—C13—C14—C15	2.0 (2)
C3—C4—C5—C9	−179.37 (13)	C12—C13—C14—C18	−177.96 (14)
C4—C5—C6—C7	−1.0 (2)	C13—C14—C15—C16	−3.1 (2)
C9—C5—C6—C7	178.25 (13)	C18—C14—C15—C16	176.82 (14)
C5—C6—C7—C2	1.3 (2)	C14—C15—C16—C11	1.0 (2)
C5—C6—C7—C10	−179.50 (13)	C14—C15—C16—C19	−178.29 (14)
C3—C2—C7—C6	−0.42 (18)	C12—C11—C16—C15	2.32 (19)
B1—C2—C7—C6	179.94 (12)	B1—C11—C16—C15	−173.84 (12)
C3—C2—C7—C10	−179.59 (12)	C12—C11—C16—C19	−178.42 (13)
B1—C2—C7—C10	0.77 (19)	B1—C11—C16—C19	5.4 (2)

### Hydrogen-bond geometry (Å, °)

**Table:**

*D*—H···*A*	*D*—H	H···*A*	*D*···*A*	*D*—H···*A*
O1—H1···Cg^i^	0.84 (2)	2.83 (2)	3.523 (2)	141 (2)

Symmetry codes: (i) −*x*, −*y*, −*z*.
